# Draft of the self-regulated learning scale items for learning nursing skills based on the cyclical phase model and evaluation of their content validity

**DOI:** 10.20407/fmj.2024-025

**Published:** 2024-12-27

**Authors:** Harumi Kato, Yumiko Miyoshi, Kimie Takehara

**Affiliations:** 1 Graduate School of Health Sciences, Fujita Health University, Toyoake, Aichi, Japan; 2 Faculty of Nursing, Fujita Health University, School of Health Sciences, Toyoake, Aichi, Japan

**Keywords:** Cyclical phase model, Item-level content validity index (I-CVI), Nursing skill, Scale development, Self-regulated learning

## Abstract

**Objectives:**

Extant studies report that self-regulated learning affects academic achievement and performance. Therefore, this study aimed to prepare a draft of a self-regulated learning scale to evaluate nursing skills learning based on the cyclical phase model, and to determine its content validity.

**Methods:**

Nine nursing students were interviewed to create an item pool. Next, focus group interviews were held with 6 nursing faculty members and 10 nursing students to refine the item pool and complete the drafting of scale items. Scores on the item-level content validity index (I-CVI) were obtained using a questionnaire survey of 11 nursing faculty members. Subsequently, an expert meeting was held to discuss the results. This study was approved by our university’s Research Ethics Review Committee.

**Results:**

From the initial 175-item pool, 216 draft items were selected. Of these, 134 items had I-CVI values of 0.78 or higher. After further evaluation in the expert panel meeting, the validity of 141 items (23 in the forethought phase, 99 in the performance phase, and 19 in the self-reflection phase) was finally confirmed.

**Conclusions:**

A draft of a self-regulated learning scale for nursing skills was created for nursing students, and the validity of its 141 items was confirmed.

## Introduction

Medical care-related needs have become more diverse and complex in recent years because of increasing sophistication and the changing attitudes of the target population. Under these circumstances, a high level of competence in nursing practice is needed^1,2^ and nursing skills must be reliably acquired to practice nursing. Studying nursing involves acquiring knowledge and undergoing extensive practice sessions where students can apply their knowledge by performing the acquired skills on simulated patients. Such exercises require independence, such as the ability to think and act on one’s own.^3^ In addition to in-class practice time, self-learning is essential for acquiring nursing skills.^4,5^ Successful students are effective in their independent learning, but the nature and content of student learning varies widely. The literature reports that the extent to which students practice self-learning skills is not significantly associated with passing or failing the skill examinations, and that students who demonstrate learning through any independent effort are likely to achieve success in passing the skill examinations.^6^ In short, self-learning is considered effective for students to acquire the core nursing skills for competent nursing practice because it enables them to think independently about their own learning methods. However, studies of university students have shown that students tend to passively undergo their university education, often unable to change their learning methods to match learning content, and that individual differences in independent learning attitudes are widening,^7–9^ indicating a decline in students’ independence. Similarly, students learning nursing may also demonstrate declining independence and struggle to acquire effective independent learning methods. Therefore, it is necessary to promote nursing students’ independent learning and help them to identify effective learning methods on their own.

Several studies of self-regulated learning, as a type of self-directed learning, have been undertaken. For instance, Zimmermann^10,11^ defines self-regulated learning as learning in which learners are active in their own learning process in terms of metacognition, motivation, and behavior. He describes three phases of learning processes: the process that occurs during learning (performance phase), the process of preparing for learning that precedes learning (forethought phase), and reacting to one’s own learning after learning (self-reflection phase).^12^ According to this cyclical phase model, autonomous cycling through these three phases promotes independent learning. Having a learning schedule and engaging in reflection after performing skills are crucial when learning nursing skills.^5,13^ Therefore, the cyclical phase model with its three phases is also effective for learning nursing skills. Ito^14^ states that learning strategies, an essential component of self-regulated learning, are concepts that include both behavioral and cognitive aspects and are situation-dependent. Therefore, research has been conducted in several learning situations. Most studies have focused on the cognitive domain, where knowledge acquisition was central (e.g., English and mathematics),^15,16^ but also in the psychomotor domain, which involves action and movement, such as physical education,^17^ and medical education.^18^ These studies report that self-regulated learning affects academic achievement and performance. Therefore, we also consider self-regulated learning as effective when learning nursing skills in the psychomotor domain. As such, promoting self-regulated learning based on the cyclical phase model for acquiring nursing skills may promote the reliable acquisition of nursing skills and improve nursing practice competence.

To promote self-regulated learning and help students acquire nursing skills, faculty need to understand students’ self-regulated learning. However, self-regulated learning is challenging to identify because learners are active in their own internalized learning processes in terms of metacognition, motivation, and behavior. Using scales can help to visualize self-regulated learning. Scales that measure learning strategies and self-regulated learning in acquiring nursing skills include the Nursing Skills Learning Strategies Scale^19^ and the Self-Regulated Learning Strategies in Acquiring Nursing Skills Scale.^20,21^ However, these scales do not incorporate the phases of the cyclical phase model of self-regulated learning, making it challenging to measure each phase of the model. A scale that measures all phases is necessary because the three phases must cycle to promote self-regulated learning. Thus, we developed a scale to measure nursing students’ self-regulated learning for acquiring nursing skills based on the cyclical phase model. We aimed to prepare a draft of a self-regulated learning scale for nursing skill learning based on the cyclical phase model, which is the first step of scale development, and to evaluate the content validity of the draft scale items.

## Definitions of terms

### 1. Self-regulated learning

Learning in which learners are active in their own learning process in terms of metacognition, motivation, and behavior.^10,11^ Self-regulated learning comprises three phases: forethought, performance, and self-reflection phases.^12^

#### a) Forethought phase

This phase consists of the motivation and task analysis that occurs before the student engages in learning. It includes goal setting, strategy planning, self-efficacy, outcome anticipation, task interest/value, and goal orientation.

#### b) Performance phase

This phase occurs during learning and affects learning directly. It includes task strategies, self-instruction, imagery, time management, environmental structuring, help-seeking, interest incentives, self-consequences, metacognitive monitoring, and self-recording.

#### c) Self-reflection phase

This phase occurs after learning and is a reaction to one’s own learning outcomes. It comprises self-evaluation, causal attribution, self-satisfaction/affect, and adaptive/defensive behaviors.

### 2. Nursing skills

Nursing skills form the basis of nursing practice and nursing action, consisting of basic movements based on scientific evidence.

## Methods

### Scale development process

**Figure 1:**
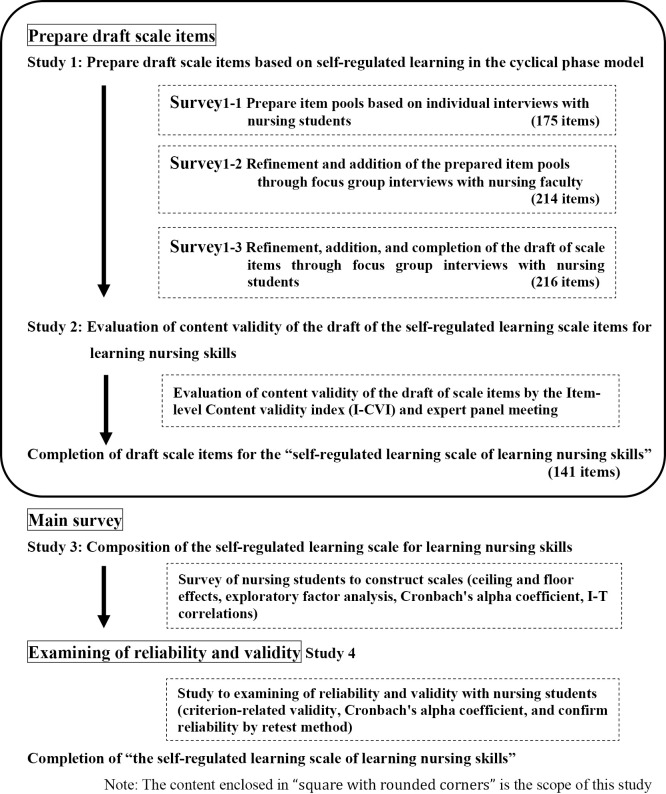
Development process of the self-regulated learning scale for learning nursing skills

The scale development process was performed as follows, in accordance with the basic process of psychometric scale development (Figure 1).^22–25^

Study 1: Prepare draft scale items based on self-regulated learning in the cyclical phase model (this study).

The draft scale items were completed using Survey 1-1 to prepare item pools based on individual interviews with nursing students, Survey 1-2 to refine and add the prepared item pools through focus group interviews with nursing faculty, and Survey 1-3 to refine and add the draft scale items through focus group interviews with nursing students.

Study 2: Evaluation of content validity of the draft of the self-regulated learning scale items for learning nursing skills (this study).

Content validity was evaluated using the item-level content validity index (I-CVI). I-CVI is a method in which experts quantitatively assess the relationship between each item of a draft scale and the concept being measured. In addition, the results of the I-CVI were evaluated by an expert panel.

Future studies

In Study 3, the scale will be constructed through exploratory factor analysis and calculation of Cronbach’s alpha coefficient. In Study 4, reliability and validity will be examined using criterion-related validity and calculation of Cronbach’s alpha coefficient, leading to the completed scale.

The present paper describes Studies 1 and 2 only.

### Study 1: Prepare draft scale items based on self-regulated learning in the cyclical phase model.

Survey 1-1: Prepare item pools based on individual interviews with nursing students.

Semi-structured interviews were conducted individually with nine first- and second-year students at two universities who had experienced an evaluation of their basic nursing skills. The participants were selected because they were primarily learning basic nursing skills at the time and could talk about their own learning. In individual interviews, the participants were asked to describe the methods they used to learn basic nursing skills, and a verbatim transcript was compiled from the data obtained. Content representing self-regulated learning was extracted according to the concepts comprising the cyclical phase model. The extracted content was classified, organized, and integrated to prepare the item pool.

Survey 1-2: Refinement and addition of the prepared item pools through focus group interviews with nursing faculty.

Focus group interviews were performed with six faculty members involved in nursing skills education and studying self-regulated learning at five universities. In the interviews, participants were shown an item pool prepared from the individual interviews with students and asked whether it represented self-regulated learning. The obtained data were examined in light of the constructs of the cyclical phase model, and items that could not be considered self-regulated learning or whose semantic content was difficult to understand were deleted or modified. Subsequently, the focus group interviews were conducted again to verify the validity of the revised and additional items in the item pool.

Survey 1-3: Refinement, addition, and completion of the draft scale items through focus group interviews with nursing students.

Focus group interviews were performed with 10 third- and fourth-year students at two universities. Focus group interviews were conducted twice, with five participants each time. The participants had completed their learning of the basic nursing skills. Therefore, they had acquired several learning methods, and had had the opportunity to perform the skills learnt on the target population. Hence, we considered they would be able to provide a wide range of opinions on the item pool. Participants were shown a refined item pool from the focus group interviews with the faculty and asked for their opinions on whether the meaning was fully comprehensible. Based on the data obtained, items whose meanings were complicated for students to understand and those unrelated to actual learning were revised or deleted.

### Study 2: Evaluation of content validity of the draft of the self-regulated learning scale items for learning nursing skills.

#### 1. Participants

The participants were 20 faculty members with experience in nursing skills education at nursing universities, sampled using convenience sampling. The evaluation of content validity using I-CVI is considered sufficient for a minimum of five participants.^26,27^ Therefore, considering the response rate and valid response rate, we targeted 20 participants.

#### 2. Data collection

We used a self-administered anonymized questionnaire survey that was mailed to the participants. The survey items included demographic data (gender, age, years of experience as a nurse, years of experience as a faculty member, and years of experience in teaching nursing skills) and the draft scale items. The concept of the cyclical phase model was shown to the participants. They were asked to rate the relationship between each concept and each questionnaire item on a four-point scale (1: not at all relevant, 2: slightly relevant, 3: quite relevant, and 4: highly relevant) in accordance with the method recommended by Polit et al.^25,28^ In addition, an open-ended question was provided to solicit opinions on the draft scale items. The data collection was conducted from January to March 2022.

#### 3. Data analysis

The I-CVI was calculated by dividing the participants who responded “4: highly relevant” or “3: quite relevant” on the four-point scale by the total number of participants. The threshold value of I-CVI for content validity was set at 0.78 or higher, as Polit et al. recommend.^25,28^ Items with an I-CVI between 0.70 and 0.78 were revised or deleted after careful consideration based on the I-CVI results and the responses to the open-ended question (expert panel meeting, henceforth). This step was performed by five faculty members engaged in research on self-regulated learning who have experience in nursing education.

## Ethical considerations

Participants were informed orally or in writing about the purpose and methods of the study, the protection of their personal information, and their voluntary participation. This study was approved by the Medical Research Ethics Review Committee of Fujita Health University (Reception No.: HM22-592).

## Results

### Study 1: Prepare draft scale items based on the concept of self-regulated learning in the cyclical phase model.

Survey 1-1: Prepare item pools based on individual interviews with nursing students.

All participants were women, and the mean age was 21.2 years. One participant had prior university education in a field other than nursing and work experience before entering nursing school. The total number of items extracted from the data was 269, with 39 in the forethought phase, 211 in the performance phase, and 19 in the self-reflection phase. After organizing and integrating the extracted content, a pool of 175 items was prepared: 20 in the foresight phase, 146 in the execution phase, and nine in the self-reflection phase.

Survey 1-2: Refinement and addition of the prepared item pools through focus group interviews with nursing faculty.

All participants were women, with an average of 12.2 years of experience in teaching nursing skills. Based on the content of the interviews, 53 items were modified: 13 in the forethought phase, 36 in the performance phase, and four in the self-reflection phase. Only two items were deleted from the performance phase. Forty-one items were added: 11 in the forethought phase, 19 in the performance phase, and 11 in the self-reflection phase. Finally, 214 items remained, consisting of 31 items in the forethought phase, 163 in the performance phase, and 20 in the self-reflection phase.

Survey 1-3: Refinement, addition, and completion of the draft scale items through focus group interviews with nursing students.

Again, all participants were women, with an average age of 20.6 years. None of the students had worked or been educated at other universities before entering their present university. As a result of the review based on the interview content, 46 items were modified: 44 in the performance phase and 2 in the self-reflection phase. Only four items were deleted in the performance phase. Six items were added: five in the performance phase and one in the self-reflection phase. Finally, 216 scale items were determined as the final draft of scale items: 31 in the forethought phase, 164 in the performance phase, and 21 in the self-reflection phase.

### Study 2: Evaluation of content validity of the draft of the self-regulated learning scale items for learning nursing skills.

#### 1. Characteristics of participants

Of the 17 respondents (85.0% response rate), data from 11 respondents who had responded to all items were included in the analysis (64.7% valid response rate). The characteristics of the participants are shown in Table 1.

#### 2. Item-level content validity index (I-CVI)

A total of 134 items had an I-CVI of 0.78 or higher: 19 in the forethought phase, 96 in the performance phase, and 19 in the self-reflection phase. The 19 items that had an I-CVI of 0.78 or higher in the forethought phase (Table 2) included “Study by setting your own intermediate goals regarding duration or images of achievement” and “Effective learning methods from the study of previous skills are used again.” However, 12 items had an I-CVI of less than 0.78, including “Learning when invited by a friend.” The 96 items that had an I-CVI of 0.78 or higher in the performance phase (Table 3) included “Observe each other performing a nursing skill and share feedback with your friend” and “Receive guidance from the faculty member regarding a skill that I am unable to execute properly.” The 68 items with an I-CVI of less than 0.78 included “Learning each manual skill (what constitutes a nursing skill) a similar number of times.” The 19 items in the self-reflection phase (Table 4) for which the I-CVI was 0.78 or higher included “After performing a nursing skill, determine whether one’s own nursing skill was adequate” and “After performing a nursing skill, consider the reasons why the manual skill (what constitutes a nursing skill) could not be performed properly.” In contrast, the I-CVI was less than 0.78 for two items, including “I am disgusted with myself for failing to perform a nursing skill correctly.”

During the expert panel meeting, we evaluated the I-CVI results for content validity. Then, we rechecked the results to ensure there was no overlap in the semantic content of the items across the entire scale. In the forethought phase, we revised the wording of 10 items, such as changing “Plan when to receive guidance from a teacher before starting learning” to “Decide when to receive guidance from a teacher in advance,” because the former was less recognizable as part of the forethought phase. In the performance phase, we revised the wording of 23 items, such as changing “Perform a nursing skill many times to learn with your body” to “Perform a nursing skill properly by repeating it many times,” which indicates the strategy to achieve the objective more clearly. In the self-reflection phase, “After performing the skill, ask a friend to judge whether the skill you performed was executed properly” was deleted because it was a repetition of other items. As a result, the content validity of 141 items was confirmed, including 23 items in the forethought phase, 99 in the performance phase, and 19 in the self-reflection phase.

## Discussion

### Validity of the process of preparing the proposed scale items

Creating the draft versions of items for this study involved interviews with students learning nursing skills and faculty members who had experience in teaching nursing skills. Thus, we were able to obtain adequate self-learning content from nursing students. Kawaguchi^23^ recommends creating an item pool with several hundred questions. We considered the 216-item pool for this study was adequate.

### Content validity of the draft scale items

The content validity examination using the I-CVI and the expert panel qualitative review confirmed the content validity of the 141 draft scale items.

In the forethought phase, the validity of items such as “Study by setting your own intermediate goals regarding duration or images of achievement,” “Determine in advance the learning methods for nursing skills that you found difficult for you to perform in class,” and “I think I will obtain a good grade if I can perform my nursing skills properly” was confirmed. These items are similar to those in existing scales, such as the Planning/Adjustment in the Nursing Skills Learning Strategies Scale^19^ and Imagination Strategies in the Self-regulating Learning Strategies in Acquiring Nursing Skills Scale.^20,21^

In the performance phase, the validity of items such as “Perform a nursing skill properly by repeating it many times,” “Focus on learning the points that require special attention when performing the skill in a practical setting,” and “Practice many times, focusing on a manual skill (what constitutes a nursing skill) that cannot be performed reliably” was confirmed. These items had been revised and included in the draft of scale items during the expert panel meeting, and were similar to a previous study in that each nursing skill had its own specific points with learners encouraged to repeat or focus on the points to accomplish the task.^29^ In the self-reflection phase, the validity of items was confirmed, such as “After performing a nursing skill, determine whether one’s own nursing skill is adequate” and “After performing a nursing skill, consider the reasons why the manual skill (what constitutes a nursing skill) could not be performed properly.” These items were similar to those in previous studies that focused on the critical role of reflection after performing a task when acquiring nursing skills.^13^ Based on the above, we can conclude that the draft scale contains content focused on acquiring nursing skills.

## Limitations and future research

Following the cyclical phase model, we prepared an item pool and confirmed its content validity. Ultimately, we were able to prepare items that were valid across all phases: the forethought, performance, and self-reflection phases. This study conducted multiple individual and focus group interviews while developing the draft scale items. Although the number of universities targeted for each individual survey was not high, over the entire process we recruited participants and collected data from several universities. Thus, the findings have some degree of generalizability. Future studies will focus on the reliability and validity of the scale, using a survey to evaluate the draft scale items obtained in this study, ultimately leading to a final version.

Nursing students can use this scale to evaluate their degree of self-regulated learning, which may be helpful for the effective learning of nursing skills. The scale can also be used by nursing faculty to evaluate their students’ degree of self-regulated learning. It can be applied as an evaluation tool to consider educational support tailored to individual students’ self-regulated learning characteristics and educational methods that promote self-regulated learning. Furthermore, it can be used to examine factors related to self-regulated learning.

## Conclusions

The scale developed in this study comprises 141 items, with 23 items in the forethought phase, 99 in the performance phase, and 19 in the self-reflection phase. These items were proposed as a draft scale to measure self-regulated learning when acquiring nursing skills, and the content validity of the items was confirmed.

## Figures and Tables

**Table1:** Characteristics of participants (n=11)

	Characteristics	n	Median	IQR	sup	inf
Gender	Men	0				
	Women	11				
Age	40–49	2				
	50–59	6				
	60 years or more	3				
Years of experience as a nurse	5–10	5	12	4.50	28	5
	11–15	5				
	16 years or more	1				
Years of experience as a faculty member	5–10	1	19	6	26	5
	11–15	2				
	16–20	4				
	21–25	1				
	26	2				
	Non-response	1				
Years of experience in teaching nursing skills	5–10	1	19	4.8	26	5
	11–15	3				
	16–20	6				
	21–26	1				

Abbreviation: IQR, Interquartile Range; sup, supremum; inf, infimum

**Table2:** I-CVI values of the self-regulated learning scale items in learning nursing skills (forethought phase)

Items	I-CVI value
Study by setting your own intermediate goals regarding duration or images of achievement.	1.00
Determine what I will learn in order to achieve my goals.	1.00
Start learning after making a plan detailing what to do and when.	1.00
Effective learning methods from the study of previous skills are used again in this study.	1.00
Determine in advance the learning methods for nursing skills that you found difficult to perform in class.	1.00
Decide how to study by taking into account other assignments and my own schedule.	1.00
I decide on study time while taking into account other assignments and my own schedule.	1.00
I think if I learn properly, I can acquire the nursing skill.	1.00
I think I will obtain a good grade if I can perform my nursing skills properly.	1.00
I value the correct implementation of nursing skills.	1.00
Learn the nursing skills to serve the target population properly.	1.00
Decide when to learn in the practice room (seminar room), taking into account the availability of the practice room (seminar room).	0.91
(omitted)	
I would be able to take the time to learn and acquire the skill.	0.73
Plan when to receive guidance from a teacher before starting learning.	0.73
Schedule my learning so that I can spend as much time as possible learning.	0.64
Decide in advance how to learn the important points.	0.64
I believe that if the skill can be performed properly, the patient or simulated patient will be pleased with it.	0.64
Share my self-learning plan with my learning partner.	0.55
Think of a place to learn skills other than the practice room (seminar room).	0.45
Learning when invited by a friend.	0.27

Selected 20 of 31 items

**Table3:** I-CVI values of the self-regulated learning scale items in learning nursing skills (performance phase)

Items	I-CVI value
Observe each other performing a nursing skill and share feedback with your friend.	1.00
Receive guidance from the faculty member regarding a skill that I am unable to execute properly.	1.00
Focus on learning the points that require special attention when performing the nursing skill in a practical setting.	1.00
Learning with a friend who can properly perform the skill.	1.00
Perform the skill by checking what you have learned in class with a friend.	1.00
Practice many times, focusing on a manual skill that cannot be performed reliably.	1.00
(omitted)	
Before actually performing the skill, imagine the operation.	0.91
Imagine a situation in which you are actually practicing nursing skills.	0.91
Learning in your free time, even for short periods.	0.91
Determine what I want to learn in advance and start learning as soon as I have time to do so.	0.91
After learning on my own, I request guidance from a faculty member.	0.91
If a manual skill performed by a friend is different from yours, ask why.	0.91
Watch the video and imitate the movement of the body.	0.91
Using the class materials, performing while confirming each one’s manual skill.	0.91
(omitted)	
If I have a scheduling conflict with my learning partner, I can study with another friend.	0.73
Depending on the content, learning will take place not only in the practice room (seminar room) but also at home or during the commute to school.	0.73
Manual skills that can be performed alone are learned alone.	0.73
Describe what I learned that day.	0.73
Perform a nursing skill many times to learn with your body.	0.73
When performing a skill for the first time, watch a video and repeat and imitate in one go.	0.64
Follow the performance and write on paper what to do and say.	0.64
(omitted)	
Just before performing the skill, one feels that one’s skill is inadequate.	0.36
Learning each manual skill a similar number of times.	0.27
Learn when there is no preparation time (e.g., changing clothes or commuting).	0.27
When a manual skill that cannot be done properly is the same as a friend’s, try to think it is not a problem if it cannot be done.	0.18

Selected 25 of 164 items

**Table4:** I-CVI values of the self-regulated learning scale items in learning nursing skills (self-reflection phase)

Items	I-CVI value
After performing a nursing skill, determine whether one’s own nursing skill was adequate.	1.00
After performing a nursing skill, consider the reasons why the manual skill could not be performed properly.	1.00
I will use the same learning methods that were effective in learning the next skill.	1.00
After performing a nursing skill, reflect on one’s own skills and identify whether manual skills were adequately performed or not.	1.00
Listen to evaluations (feedback) from the faculty members and instructors to judge whether our skills were adequately performed.	1.00
Reflect on what the patient or simulated patient said or did to judge if our skills were adequately performed.	1.00
After actually performing the skill, reflect on what led to proper performance.	1.00
After performing the skill, reflect on which aspect needed the most time.	1.00
Listen to evaluations (feedback) from the faculty members and instructors and reflect on why such an evaluation was made.	1.00
Improve learning methods for subsequent learning.	1.00
After performing the skill, reflect on why it took so long to perform it.	1.00
If you receive feedback from others you disagree with, try to think about what it means.	1.00
(omitted)	
Feel satisfied with the reflection and self-assessment after actually performing the skill.	0.91
After performing the skill, ask a friend to judge whether the skill you performed was executed properly.	0.73
I am disgusted with myself for failing to perform a nursing skill correctly.	0.45

Selected 15 of 21 items
